# Deuteration Effects on the Physical and Optoelectronic Properties of Donor–Acceptor Conjugated Polymers

**DOI:** 10.1021/acs.macromol.4c02778

**Published:** 2025-04-29

**Authors:** Kundu Thapa, Madison Mooney, Guorong Ma, Zhiqiang Cao, Gage T. Mason, Naresh Eedugurala, Surabhi Jha, Derek L. Patton, Jason D. Azoulay, Simon Rondeau-Gagné, Xiaodan Gu

**Affiliations:** † School of Polymer Science and Engineering, The University of Southern Mississippi, Hattiesburg, Mississippi 39406, United States; ‡ Department of Chemistry and Biochemistry, University of Windsor, Windsor, Ontario N9B 3P4, Canada; § School of Chemistry and Biochemistry, Georgia Institution of Technology, Atlanta, Georgia 30332-0002, United States

## Abstract

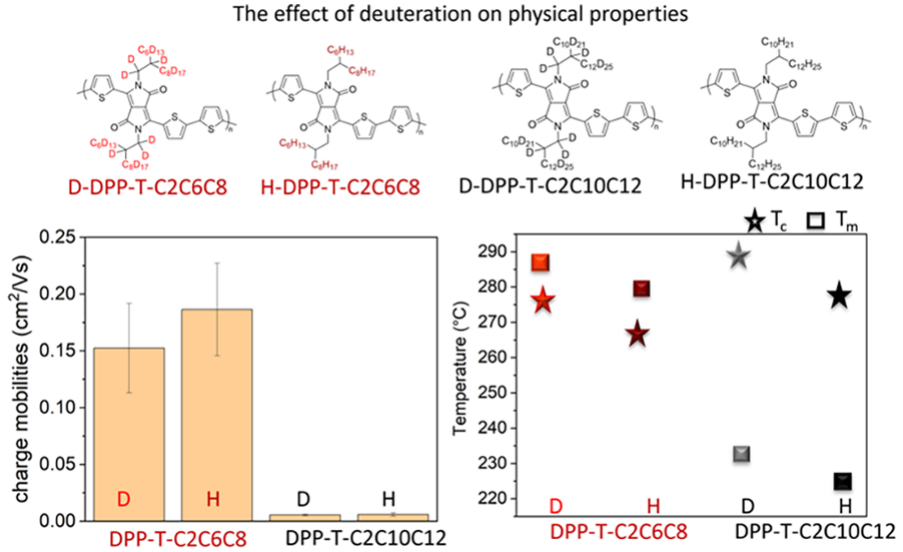

The significant differences in scattering cross sections between deuterium and protium are unique to neutron scattering techniques and have been a long-standing area of interest within the neutron scattering community. Researchers have explored selective deuteration to manipulate scattering contrast in soft matter systems, leading to the widespread use of deuterium labeling in materials development. As deuteration changes the atomic mass, it alters physical properties such as molecular volume, polarizability, and polarity, which in turn may affect noncovalent interactions and crystal ordering. Despite previous studies, there remains a limited understanding of how deuteration impacts donor–acceptor (DA) conjugated polymers. To address this, we synthesized deuterated DPP polymers and systematically investigated the effects of side-chain deuteration on their thermal stability, crystal packing, morphology, and optoelectronic properties. We found that deuteration increased the melting and crystallization temperatures of DPP polymers, although it did not significantly alter their morphology, molecular packing, or charge mobility. These properties were assessed by using atomic force microscopy (AFM), X-ray scattering, and thin-film transistor device measurements, respectively, for DPP polymers. Our work shows that deuterium labeling could be a powerful method for controlling scattering length density, enabling neutrons to study the structure and dynamics of conjugated polymers without impacting their electronic performance.

## Introduction

Selective deuteration, when paired with neutron scattering, serves as a powerful method for characterizing functional materials because of the significant contrast in coherent scattering length density between deuterium (6.67 × 10^–15^ m) and protium (−3.37 × 10^–15^ m).^1^ This stark contrast enables a detailed analysis of specific components within a polymer chain.^2−6^ Since soft matter frequently contains a high amount of hydrogen, selective deuteration has become an essential technique for adjusting the scattering contrast in neutron scattering experiments.^7−9^

Neutron scattering, coupled with deuteration, is extensively used to uncover molecular details of the structure and dynamics of soft matter due to its nanoscale resolution.^1,10,11^ This technique has made significant contributions to fundamental research in polymeric materials and has become a crucial tool in the field of organic electronics.^8,12−15^ For instance, Cotton et al. demonstrated the conformations of polystyrene molecules in the bulk amorphous state using deuterium labeling and SANS, which was impossible to measure directly with other instruments such as X-ray and light scattering.^16,17^ Following this, several studies focused on polymer structure, including Russell et al.*’s* work, which employed SANS to investigate the phase behavior of poly(3-pentylthiophene) (P3PT) in both as-spun and thermally annealed thin films.^18,19^ Apart from structural studies , Gomez et al. studied the dynamics of these conjugated polymers, which revealed the impact of side-chain dynamics on the backbone.^20^ Similarly, Pozzo et al. applied deuteration strategically to study the temperature-dependent dynamics of P3HT by isolating the motions of specific polymer components.^21^ Specifically, they used partially deuterated P3HT to reduce the incoherent scattering contribution from the side chains and to highlight the backbone dynamics, which are critical for charge transport properties. Later on, the decoupling of side-chain versus backbone conformations of P3ATs was demonstrated through neutron scattering combined with selective deuteration.^22^ By selectively deuterating the side chains to enhance its contrast, the backbone conformations were deconvoluted, revealing differences in rigidity between the backbone alone versus the whole polymer chain in a fully dissolved state.^22,23^ These studies highlighted the importance of deuteration in distinguishing rigidity in different parts of the polymer chain (backbone versus side chains) to fine-tune charge transport properties. In subsequent research, Pozzo et al. investigated the solid-state structure of the blend by using a mixture of deuterated commodity polymer as a matrix and protonated conjugated polymers.^24^ They combined neutron and X-ray scattering to quantitatively and qualitatively explore the impact of molecular structure, blend composition, and processing conditions in the final morphology and performance of the polystyrene and CPs blend.^24^ This research highlighted the importance of deuteration in understanding the phase morphology of blends in bulk states via deuterium labeling and neutron scattering to optimize the electronic performance of organic electronic devices. More recently, deuterated ladder polymer has also been synthesized by the Fang group.^25^ Deuterating the side chains in ladder polymers is a promising method for uncovering the complex backbone structure.

Initially, deuterated and protonated polymers were presumed to be identical, but several studies have been reported since the early 1970s confirming that deuteration can slightly influence polymer properties, such as melting and crystallization behavior.^26,27^ For instance, deuterated polyethylene exhibits a lower melting temperature than its protonated counterpart, as discussed by Stehling et al.^27^ In the 1980s, Bates et al. demonstrated that deuteration lowers the melting temperature of nonpolar polymers, such as polyethylene, polystyrene, and polypropylene, due to reduced polarizability.^26^ The key difference lies in the fact that the atomic mass differences between deuterium and protium result in variations in the polarizability and molecular volume of the bonds throughout the polymer.^28^ Additionally, the shorter bond length of C–D compared to C–H suggests that C–D sites exhibit greater oxidative stability.^6^ This slight difference in bond length also affects intermolecular interactions.^29^ Recent investigations by Hong et al. showed that selective deuteration resulted from weaker C–D bond polarizability, leading to reduced crystallinity and melting temperature.^30^ A follow-up study by Hong et al., using differential scanning calorimetry (DSC), revealed that increasing deuteration content in poly(ε-caprolactone) (PCL) lowers both crystallinity and melting temperatures.^31^ The lowering of the melting temperature was attributed to the variation in the weak hydrogen-bond-like intramolecular interaction. Through this study, they highlighted that the crystallization kinetics are sensitive to the nature of deuteration sites.^31^

While significant research has been conducted on traditional commodity polymers, studies on the effect of deuteration on conjugated polymers remain limited. Conjugated polymers are crucial in semiconductor devices, including thin-film transistors (TFTs),^32^ light-emitting diodes (LEDs),^33^ solar cells,^34,35^ and bioelectronics.^36,37^ Poly(3-alkylthiophene), a widely studied conjugated polymer, is known for its excellent electrical and mechanical properties.^38−41^ However, there has been limited research on how deuteration affects the crystallization, melting, and optoelectronic properties of poly(3-alkylthiophenes) (P3ATs).^42−45^ For instance, Xiao et al. investigated the effects of deuteration on poly(3-hexylthiophene) (P3HT) by strategically deuterating the main backbone and side chains, revealing a significant reduction in absorption and current density due to main-chain deuteration, as shown by UV–vis and thin-film device studies.^43^

Only recently, Sumpter et al. explored the influence of deuteration at various sites of P3HT on its crystallinity.^45^ Similarly to nonpolar polymers, here they discovered that the crystallinity was lowered after deuteration, which is dictated by the deuteration sites.^45^ As deuteration on the main-chain backbone had the most drastic impact, lowering the crystallinity, they attributed this reduction in crystallinity to the difference in quantum nuclear effects due to changes in zero-point vibrational energy and dynamic correlation of the dipole fluctuations.^45^ Recently, donor–acceptor (D–A) polymers have gained attention for their superior electronic properties, attributed to their complex high conjugation and the presence of fused acceptor and donor units in the backbone, enabling remarkable charge transport properties that surpass even those of amorphous silicon semiconductors.^46−49^ However, the effects of deuteration on D–A conjugated polymers remain unexplored, necessitating further research to understand these influences on their physical properties.

In this work, we investigated the impact of side-chain deuteration on the thermal, crystallization, and optoelectronic properties of diketopyrrolopyrrole (DPP) D–A CPs. We synthesized deuterated DPP polymers and systematically investigated the effects of side-chain deuteration on their thermal stability, crystal packing, morphology, and optoelectronic properties. Notably, deuteration resulted in increased melting and crystallization temperatures in the DPP polymers. However, deuteration did not significantly affect the morphology, molecular packing, or charge mobility, as determined through atomic force microscopy (AFM), X-ray scattering, and thin-film transistor device measurements for DPP polymers. This work provides an answer to the neutron scattering community regarding the potential impact of deuteration on functional semiconductive D–A polymers.

## Experimental Section

### Materials

The synthesis of all DPP and P3AT polymers was performed following previously reported procedures.^22,23^ Concurrently, the protonated and deuterated side chains were synthesized according to the previous report.^23^ Briefly, the deuterated side chains were first synthesized through a hydrogen–deuterium exchange reaction using a high-pressure Parr reactor, followed by the subsequent insertion of deuterated and protonated side chains to DPP through alkylation.^23^ Then, the DPP building block was polymerized with a stannylated thiophene monomer through a Stille cross-coupling reaction. Detailed information regarding the synthetic methodology and corresponding NMR spectra of the polymer has been reported previously.^23^ The cyclopentadithiophene (CDT) polymers were prepared following previous reports.^50,51^ Deuteration levels of the side chains were characterized by NMR to ensure over 99% purity. High purity is needed to ensure negligible influence on the stability and properties of the polymer crystals studied here.^45^

## Characterization

### Thermal Gravity Analysis

The thermal behavior of P3PT, DPP, and CDT polymers was analyzed by using a Mettler Toledo TGA under a nitrogen atmosphere. Samples weighing between 2 and 5 mg were carefully placed in ceramic crucibles for the analysis. A constant heating rate of 10 °C/min was maintained as the temperature gradually increased from 25 to 600 °C. The temperature at which a 5% mass loss was observed was recorded as the degradation temperature. Each sample was replicated at least 3 times to obtain mean values and to determine the uncertainty between measurements using the standard error.

### Differential Scanning Calorimetry

DSC analysis was conducted using a Mettler Toledo DSC 3+ equipped with an FRS6 sensor under a dry nitrogen purge at a flow rate of 50 mL/min. Samples weighing between 2 and 7 mg were placed in aluminum pans with lids, and a small vent port was created by opening the lid. DPP polymers were examined over a temperature range of −90 to 350 °C, with all scans performed at a rate of 10 °C/min. The melting temperature was determined from the onset of melting in the second heating curve, while the crystallization temperature was identified from the exothermic peak in the cooling curve. Similarly to the TGA measurements, DSC analysis for each sample was replicated 3 or more times across various batches, and the mean value is provided along with the standard error.

### Dynamical Mechanical Analysis

DMA of deuterated DPP polymers was performed using a TA Instruments Q800. The glass transition temperature (*T*_g_) was identified from the peak temperature of the Tan δ curve. All experiments were carried out at a constant frequency of 1 Hz, with the temperature ranging from −90 to 300 °C and ramped at a heating rate of 3 °C/min. Sample preparation followed the method detailed in previous literature, where samples were obtained by drop-casting a 10 mg/mL solution and then formed into micrometer (μm)-thick rectangular bars.^34^

### Wide-Angle X-ray Scattering

A WAXS study was conducted using a laboratory beamline system (Xenocs Inc. Xeuss 2.0) with a copper source (*E* = 8.04 keV). To minimize air scattering, the sample chamber was maintained under vacuum throughout all experiments. Samples were kept in borosilicate glass capillaries, and the temperature was controlled by a Linkam stage THMS600. Initially, samples were characterized at room temperature and then heated up to 350 °C. For each measurement temperature, the sample was held at that temperature for 1 h to ensure complete thermal stabilization before measurement. At 350 °C, samples were exposed to X-rays for 1 h to obtain a fully melted polymer scattering profile. Diffraction images were captured using a Pilatus 1M detector (Dectris Inc.). Each data point was run for 1 h, and the data were processed using the Nika software package and WAXStools. The peak values and full width at half maximum (fwhm) are fitted by a Gaussian function using IgorPro 9.0 software.

### Atomic Force Microscopy

Surface morphology was examined using an Asylum S AFM in tapping mode. Samples were prepared by spin-coating 10 mg/mL polymers in chlorobenzene at 1000 rpm onto silicon substrates, resulting in DPP thin films. The samples were imaged in air. Each sample was replicated 3 times, with 3–6 spots chosen within a 1 μm by 1 μm radius to provide the average root-mean-square roughness (RMS).

### UV–vis Spectroscopy

UV–vis-NIR spectra were recorded by using a Cary 5000 Series UV–vis-NIR spectrometer from Agilent Technologies. All sample solutions were prepared in dichlorobenzene at a very low concentration and placed in quartz cuvettes. The experiment was conducted using Scan software, and the data were analyzed with OriginPro. Calculation of the direct band gap was performed using absorption obtained from UV–vis spectroscopy and the general equation of the Tauc plot;^52^ (α*h*ϑ)^γ^ = *K*(*h*ϑ – *E*_g_), where α is the absorption coefficient, *h*ϑ refers to photon energy, *K* refers to constant (2 for this calculation), and *E*_g_ corresponds to the band gap energy.

### TFT Device Fabrication and Device Testing

The TFT device was fabricated using a bottom-gate, top-contact geometry. A p-type silicon wafer with a resistance of 1 × 10^–3^ – 5 × 10^–3^ Ω and a 500 nm SiO_2_ layer was used as the bottom gate electrode and dielectric layer, respectively. The silicon wafer was first treated with UV/ozone for 5 min, then cut into 1.5 × 2.1 cm pieces. A polymer solution was spin-coated onto these pieces at 1000 rpm for 1 min. Gold electrodes, serving as the source and drain for the top-contact bottom-gate (TC-BG) devices, were then thermally evaporated onto the wafers using a shadow mask. The device channel width was set to 1 mm, with channel lengths of 30, 40, 50, 60, and 80 μm were fabricated. Device testing was conducted inside a glovebox on a probe station (Signatone 1160 series), with raw data collected using a Keithley 4200 semiconductor testing system. All films were annealed at 150 °C for 1 h inside the glovebox before device testing. The charge mobilities of the DPPs were calculated from the slope of the linear regime in the transfer characteristics of the device.

### Ultraviolet Photoelectron Spectrometer (UPS)

The experiments were performed using a Thermo Fisher ESCALAB 250Xi Ultraviolet Photoelectron Spectrometer with a Helium I (He I) source. The polymer was dissolved in chlorobenzene at a concentration of 10 mg/mL. The polymeric thin-films were made on silicon wafers by spin-casting at 2000 rpm for 1 minute. Thin films ranging from 50 to 90 nm were used for the UPS study. The results were further calculated using the equation below. *E*_v_ = *hv* – *E*_KE_, where, *E*_V_ is the valence band energy, and *hv* and *E*_KE_ are the energy of the photon and kinetic energy. The energy of the photon used in this study was 21.2 eV.^53^

## Result and Discussion

### Impact of Deuteration on the Thermal Mechanical Properties of Conjugated Polymers

**Figure 1:**
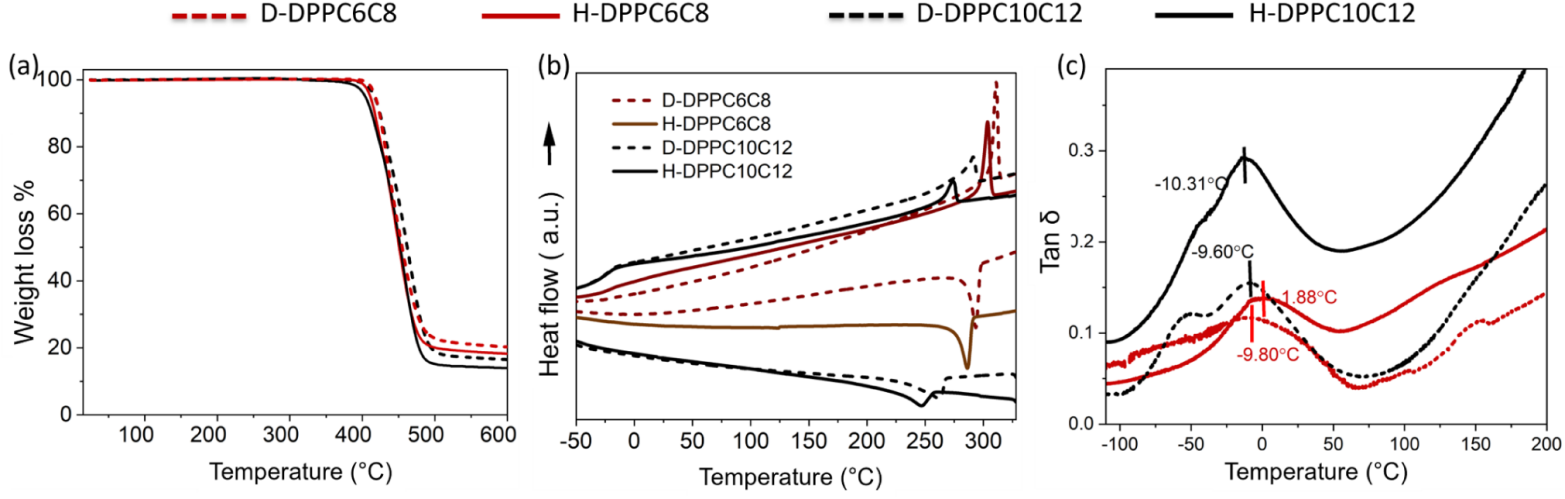
(a) TGA thermograms of all DPP polymers. (b) DSC of DPPs with second heating and cooling curves in stack plots. (c) The tan delta curves from DMA of all DPP polymers. For all parts (a), (b), and (c), the dotted line represents the deuterated polymer, and the solid line represents the protonated polymers.

We began by investigating the impact of deuteration on the thermomechanical properties of DPP polymers, since replacing hydrogen with deuterium affects the van der Waals interactions between carbon and deuterium.^1^ We analyzed two pairs of DPP polymers with different deuterated and nondeuterated side chains using TGA. The degradation temperature, defined as the point where a 5% mass loss occurs, was determined for each sample. As shown in Figure 1a, the TGA curves indicate that the deuterated D-DPP-T-C2C6C8 exhibited a slightly higher degradation temperature of 409.1 °C compared to the protonated H-DPP-T-C2C6C8 polymers, which degraded at 407.2 °C. A similar trend was observed for DPPs with longer side chains, such as D-DPP-T-C2C10C12 and H-DPP-T-C2C10C12, as listed in Table 1. The higher degradation temperature of D-DPP-T-C2C6C8 is attributed to the greater bond dissociation energy of the C–D bond (341.4 kJ/mol) compared to the C–H bond (338 kJ/mol).^1,54^ Additionally, the C–D bond length is approximately 0.005 Å shorter than the C–H bond.^26^ As for the side-chain length effect, the longer side-chain length induces a more pronounced increment compared to the short side-chain length DPP polymer, which could be attributed to the higher deuterium content in the longer side-chain DPP-T-C2C10C12 as compared to the DPP-T-C2C6C8, which increased the overall thermal stability.

**Table 1:** Summary of Thermal Measurements of DPP Polymers^a^

Polymer	*M*_n_ (kDa)	*M*_w_ (kDa)	*T*_d_ (°C)	*T*_m_ (°C)	*T*_c_ (°C)	Δ*H*_fus_ (J/g)	*T*_g_^a^ (°C)
D-DPP-T-C2C6C8	49.1	836	409.1 ± 7.4	289.0± 1.5	276.9 ± 0.7	14.3 ± 0.5	–9.80 ± 3.5
H-DPP-T-C2C6C8	34.4	475	407.2 ± 2.6	280.1 ± 5.3	269.8 ± 2.6	14.3 ± 0.4	1.88^60^ ± 0.01
D-DPP-T-C2C10C12	28.6	150	410.8 ± 5.3	252.7 ± 1.1	259.3± 0.1	11.3 ± 1.2	–9.60 ± 4.67
H-DPP-T-C2C10C12	32.4	149	403.4 ± 0.5	243.3 ± 2.5	249.4± 0.1	9.8 ± 0.7	–10.31^60^ ± 1.45

a *T*_m_ and *T*_c_ are obtained as onset and peak values, respectively. The enthalpy of fusion (Δ*H*_fus_) was calculated from the crystallization peaks. The *T*_g_ reported here for the backbone of DPP polymers, which has molecular weight of 71.7, 88.5, 73.0, and 60.6 kDa from top to bottom in Table 1.^58^

DSC analysis was conducted on the DPP polymers to examine their melting and crystallization behaviors, as shown in Figure 1b. The mean onset temperatures, along with the standard error of melting and crystallization, are listed in Table 1. To avoid the effects of molecular weight on the melting and crystallization behaviors of DPPs, we ensured that the protonated and deuterated polymers had comparable molecular weights, as shown in Table 1 unless otherwise noted. The mean melting temperature of D-DPP-T-C2C6C8 was 289.0 °C, which is 1.60% higher than that of H-DPP-T-C2C6C8. For the longer side chain, the melting temperature of deuterated D-DPP-T-C2C10C12 was 1.82% higher than that of protonated H-DPP-T-C2C6C8. This increment in melting behavior with deuteration contrasts with previous observations, where deuteration was found to lower the melting temperature in several nonconjugated polymers, such as polyethylene, polystyrene, and poly(ε-caprolactone), due to reduced polarizability.^26,31^ As the Sumpter group demonstrated, dipole interactions were the main factor affecting the crystallinity and ordering of polythiophenes, but not the molecular mass.^45^ In addition, a previous study by the Hong group on poly(ε-caprolactone) reported that the presence of oxygen atoms in the carbonyl groups, participating in weak hydrogen (H)-bond-like interactions, disrupts with deuteration, which significantly reduces with deuteration.^31^ The C–D bond has reduced polarity compared to the C–H bond; we hypothesized that the melting and crystallization temperatures would be reduced with deuteration for the DPP polymers.^43^ However, the DPP polymers did not follow such a trend. As DPP polymers are rigid with carbonyl groups that promote π–π stacking and stronger intermolecular interactions,^55^ these interactions were expected to weaken and lower the melting and crystallization temperatures with deuteration. Hence, there might be another factor involved in such an unusual increase in melting and crystallization. These rigid polymers are more planar, and planarization is known to control the chain organization, which can alter the melting temperature of the polymers through stronger molecular interactions.^56,57^ As the increment in melting and crystallization is observed for DPP polymers, unlike non-planar polymers such as PCL and polythiophenes.^30,31,45^ We speculate that the high planarity of DPP polymers could be the factor behind the unusual thermal behaviors with the deuteration. This is further evaluated through X-ray scattering studies.

Isothermal crystallization studies provide valuable insights into the crystallization behavior of conjugated polymers by calculating the enthalpy of fusion from either endothermic or exothermic peaks.^59^ All DPPs were initially heated from −90 to 350 °C to remove any residual thermal history. The enthalpy of fusion (Δ*H*_fus_) was determined from the area under the exothermic peak in the second heating curves, and the mean value of 3 replicates is provided. As shown in Table 1, the enthalpies of fusion for deuterated and protonated polymers were nearly identical, especially for the shorter side-chain DPP-T-C2C6C8. For instance, deuterated DPP-T-C2C6C8 had a mean enthalpy of fusion of 14.3 ± 0.5 J/g, compared to 14.3 ± 0.4 J/g for protonated DPP-T-C2C6C8 polymers. Similarly, deuterated and protonated DPP-T-C2C10C12 exhibited comparable enthalpies of fusion within the error bar. In addition, the melting and crystallization peaks were fitted using a Gaussian function (shown in Figures S5–S7), and the FWHM values are provided in Table S8. The obtained FWHM for both melting and crystallization decreased with deuteration for the shorter side-chain DPP polymer, whereas the opposite trend was observed for the longer side-chain DPP polymer, as shown in Table S8.

To investigate whether these changes were correlated with the dispersity (PDI), we further examined PDI variations. The shorter side-chain polymers exhibited a much higher PDI than the longer side-chain polymers but still had a lower FWHM, suggesting that this effect is not related to PDI. Additionally, the PDI values for both DPP-T-C2C6C8 and DPP-T-C2C10C12 were higher in the deuterated polymers. Thus, FWHM differences between the deuterated (D) and protonated (H) polymers studied here cannot be attributed to the PDI variations. The increased FWHM observed for the longer side-chain D-DPP-T-C2C10C12, compared with DPP-T-C2C6C8, is attributed to the increased steric hindrance of the side chain rather than a mass effect. This conclusion is supported by mass calculations, which show that deuteration increased the mass of both DPP-T-C2C6C8 and DPP-T-C2C10C12 by 8.01% and 9.36%, respectively. If the decrease in FWHM for the DPP-T-C2C6C8 polymer upon deuteration were due to a mass effect, then its FWHM should have increased with deuteration, but this is not the case. Similarly, DPP-T-C2C10C12 should have shown a decrease in FWHM with deuteration, as the molecular weight of the deuterated polymer was lower than that of the protonated one.

The impact of deuteration on *T*_g_ has not been thoroughly explored. Therefore, DMA was used to investigate how side-chain deuteration affects the glass transition behavior of the DPP polymers. We conducted DMA on deuterated DPP polymers and compared the results to the protonated DPP polymers with comparable molecular weights from our previous work in 2021.^58^ The error bars for *T*_g_ are obtained through the fitting of the Tan δ curves. As shown in Table 1, the *T_g_* values for H-DPP-T-C2C6C8 and D-DPP-T-C2C10C12 were determined from the peaks of the tanδ curves, which are shown in Figure S9. The two tanδ peaks correspond to the *T*_g_ of the side chain and backbone, from lower to higher temperature, respectively. The backbone *T*_g_ for the H and D versions of DPP-T-C2C6C8 was 1.88 °C and −9.80 °C, respectively. Similarly, the H and D versions of DPP-T-C2C10C12 exhibited *T*_g_ values of −10.31 °C and −9.60 °C, respectively. The *T*_g_ of H-DPP-T-C2C6C8 is notably higher compared to other DPPs, likely due to its much higher molecular weight, which could have significantly increased the *T*_*g*_. When the molecular weights are comparable, the *T*_g_ values before and after deuteration are similar. From these thermal studies, we concluded that although deuteration does not significantly affect *T*_g_, it does influence the melting and crystallization temperatures.

### Impact of Deuteration on the Morphology of Conjugated Polymers

**Figure 2:**
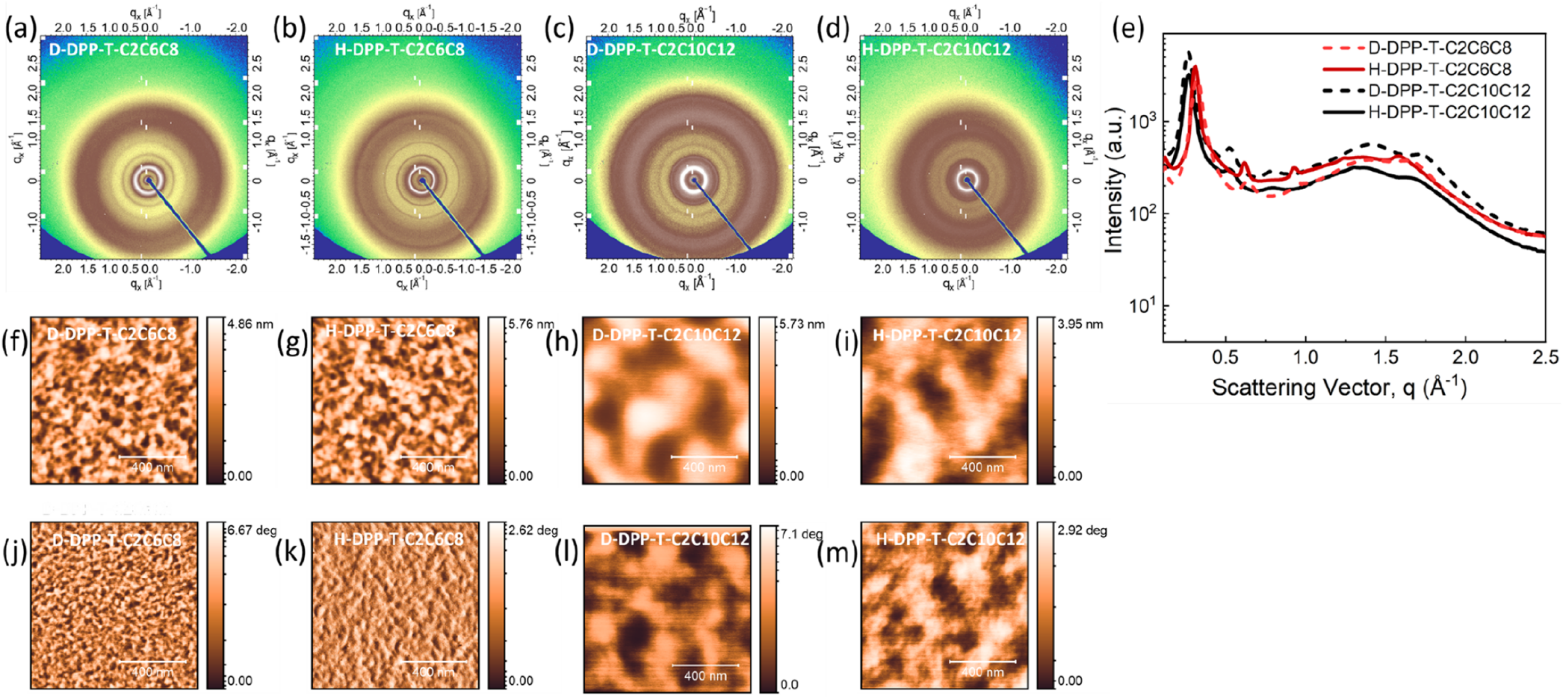
2D (a–d) and 1D (e) X-ray scattering plots for DPP polymers. AFM images of solid-state DPP thin films were obtained by spin-coating (10 mg/mL) in chlorobenzene (f–m). Top row represents the height images, whereas bottom row represents the phase angle images of DPP polymers.

Atomic force microscopy was used to examine the effects of deuteration on the morphology of the polymers. Figure 2 displays height images (f, i) and phase angle images (j, m) for the DPP polymers. We hypothesized that side-chain deuteration in DPP polymers would not change morphology because it mainly affects mass and segmental interactions. In contrast, based on previous reports, the main-chain deuteration is more likely to influence crystalline packing and optoelectronic properties by altering the conjugation length.^43^ This hypothesis was confirmed by the absence of visible differences between the morphologies of the protonated (H-DPPs) and side-chain deuterated (D-DPPs) counterparts. The DPP solutions were prepared in chlorobenzene, which was expected to exhibit some aggregated features at room temperature, particularly for DPP-T-C2C10C12. However, the AFM images revealed no visible aggregate features on the micrometer scale. Interestingly, the morphologies of the deuterated and protonated DPPs were remarkably similar, indicating that side-chain deuteration did not result in any significant changes in overall surface morphology and RMS roughness. The mean RMS roughness can be found in Table S10. Upon further examination of the DPP thin films using AFM, a lack of significant crystalline packing was observed.^43^

WAXS was employed to further investigate crystal packing, which is crucial for understanding morphology and the effects of deuteration on the molecular arrangement. The X-ray scattering patterns obtained at room temperature are shown in Figure 2. The *d*-spacing of the peaks is expressed as 2π/q. WAXS provides crystal packing information for bulk polymers, and the absolute degree of crystallinity was calculated as described in the previous publication.^60^ For these calculations, all polymers were analyzed both below and above their respective melting temperatures. The peak values were fitted by a Gaussian function.^61^Figure 2 shows the 2D (a–d) and 1D (e) scattering intensity patterns at room temperature. The absolute crystallinity values of the deuterated and protonated versions of DPP-T-C2C6C8 and DPP-T-C2C10C12 were 60%, 58%, 65%, and 55%, respectively, as shown in Table 2. Interestingly, there is a slight increment in crystallinity for both the short side-chain DPP-T-C2C6C8 polymer and the long side-chain DPP-T-C2C10C12 polymer after deuteration. Notably, the peaks at 19.5 Å and 19.8 Å correspond to the lamellar *d*-spacing for deuterated and protonated DPP-T-C2C6C8, respectively. These structural studies suggest that deuteration may not significantly affect electronic properties, which are known to be influenced by crystal packing. As the side-chain length increased, an increase in interlayer *d*-spacing was observed. For the DPP polymer with longer side chains (DPP-T-C2C10C12), both deuterated and protonated versions exhibited an interlayer *d*-spacing of 23.3 Å. This larger *d*-spacing for the longer side-chain DPP indicates weaker interlinking between the side chains and the main conjugated backbone.^62^ Additionally, the π–π stacking distances for deuterated and protonated DPP-T-C2C6C8 were 3.76 Å and 3.78 Å, respectively, while DPP-T-C2C10C12 showed π–π spacing of 3.59 Å and 3.66 Å for the deuterated and protonated versions, respectively.^62,63^ Although, the π–π peaks were similar, the FWHM obtained for π–π peaks decreased with deuteration, especially for longer side-chain DPP-T-C2C10C12 polymers. In addition, the FWHM of lamellar peaks increased slightly with deuteration for shorter side-chain DPP-T-C2C6C8, even though changes in the absolute degree of crystallinity are (∼1.03×) small for these polymers. The absolute degree of crystallinity is lowered by a factor of 1.18 for longer side-chain DPP-T-C2C10C12 with a higher deuteration content. Hence, deuteration alters the crystalline properties of the DPP polymers.

**Table 2:** Crystallographic Parameters and Surface Roughness for Deuterated and Protonated DPP Polymers, Obtained through X-ray Scattering and AFM, Respectively

Polymer	Lamellar spacing (Å)	Lamellar peak FWHM	π–π spacing (Å)	π–π peak FWHM	Absolute crystallinity (%)	Roughness (Å)
D-DPP-T-C2C6C8	19.50 ± 0.01	0.060 ± 0.001	3.76 ± 0.09	0.25 ± 0.05	60	1.89 ± 0.16
H-DPP-T-C2C6C8	19.80 ± 0.01	0.030 ± 0.001	3.78 ± 0.01	0.28 ± 0.07	58	2.31 ± 0.21
D-DPP-T-C2C10C12	23.30 ± 0.01	0.054 ± 0.001	3.59 ± 0.01	0.17 ± 0.05	65	0.55 ± 0.05
H-DPP-T-C2C10C12	23.30 ± 0.01	0.051 ± 0.001	3.66 ± 0.02	0.30 ± 0.07	55	0.61 ± 0.04

### Impact of Deuteration on the Optoelectronic Property of Conjugated Polymers

**Figure 3:**
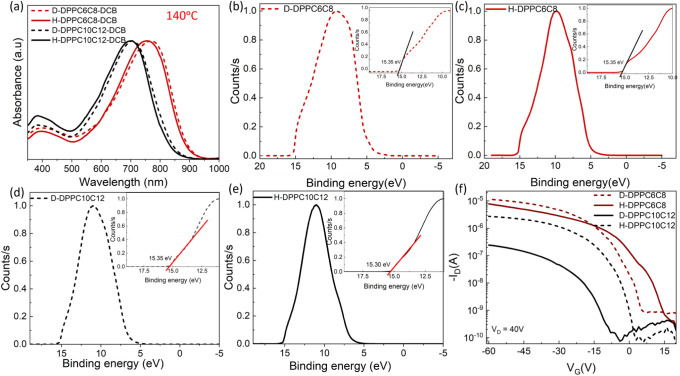
(a) UV–vis spectroscopy under fully dissolved conditions. UPS spectroscopic plots (b-e) for deuterated and protonated DPP thin-films studied via He I lamp (21.2 eV). The inset plot indicates the linear extrapolated value of valence band energies. (f) The transfer characteristics of top contact/bottom gate devices of DPP-T-C2C6C8 and DPP-T-C2C10C12 are shown. The applied drain voltage was 40 V.

In this study, we explored how deuteration affects π–π interactions in both fully dissolved (140 °C) and aggregated states (25 °C) for both solution and thin-film forms using UV–vis spectroscopy. Figure 3 displays the UV–vis spectrum at 140 °C in dichlorobenzene (DCB), where elevated temperatures and a halogenated solvent were used to fully dissolve the DPP polymers. It is well known that aggregation behavior significantly influences the electronic performance of polymers.^64^ The strong donor–acceptor interactions and π–π interactions in high-performance D–A CPs promote aggregate formation.^35,65,66^ Additionally, intra- and interchain interactions within polymers are critical factors affecting electronic performance. Deuteration is known to modify noncovalent interactions due to changes in molecular volume and polarizability.^43,67^ Recent studies have shown that deuteration alters the light absorption behavior of P3HT, resulting in much weaker absorption compared to its protonated counterparts.^43^

The absorption profile of the DPP polymer revealed dual absorption bands, with the peak at 700–800 nm corresponding to the π–π interchain interactions in DPP polymers.^68^ When fully dissolved, D-DPP-T-C2C6C8 and H-DPP-T-C2C6C8 showed peaks at λ = 793 nm and λ = 783 nm, respectively, indicating a negligible energy shift (1.9 × 10^–2^ eV). The maximum absorption peak is attributed to the HOMO–LUMO transition. Similarly, a negligible red shift (8 × 10^–3^ eV) was observed in the π–π interaction peaks of deuterated and protonated DPP-T-C2C10C12, which could be due to increased planarity.^30^ Across both solution and solid states, deuteration resulted in only minor changes (0.2–1.2%) in absorbance peak shifts, suggesting that deuteration does not significantly affect aggregation behavior in either state. The observed blue-shifting (for example, λ_max_ = 696 nm for H-DPP-T-C2C6C8 to λ_max_ = 629 nm for H-DPP-T-C2C10C12) with longer side chains in DPP polymers aligns with previous reports and is attributed to the change in backbone planarity with increasing length of the side chain.^69^

While this study primarily focused on the effects of deuteration under fully dissolved conditions, we also examined these DPPs at room temperature in both aggregated solution states and thin-film states. A table summary is provided in the supplementary Table S14. At room temperature, longer side-chain DPPs exhibited aggregated features, as shown in Figure S12. In the solid state, the peak absorbance of D- and H-DPP-T-C2C6C8 showed aggregation peaks centered at λ = 832 nm and λ = 830 nm, respectively. A slight energy shift (Δ*h*ν = 3.6 × 10^–3^ eV) was observed between D- and H-DPP-T-C2C10C12 in the solid state. In solution, the aggregation peaks (0–0) of D/H-DPP-T-C2C6C8 appeared at λ = 847 and 842 nm, respectively. These peaks were blue-shifted for DPPs with longer side chains in solution; for example, D/H-DPP-T-C2C10C12 showed peaks at λ = 822 nm and λ = 813 nm, respectively, with a blue shift of Δ*h*ν = 1.7 × 10^–2^ eV. The band gap was estimated using the Tauc plot method, with calculated values of 1.25 eV for D-DPP-T-C2C6C8 and 1.26 eV for H-DPP-T-C2C6C8, indicating a negligible change in the optical band gap after deuteration.^52^ Similarly, minimal changes were observed in the longer side-chain DPP polymers (DPP-T-C2C10C12) upon deuteration.

The occupied electronic states were directly measured using UPS, as shown in Figure 3b,e, with a helium light source (He I, 21.2 eV). The obtained HOMO levels for both D/H-DPP-T-C2C6C8 were −5.85 eV, consistent with literature values, indicating that deuteration does not affect HOMO levels.^62,63^ The LUMO levels, calculated by combining UV–vis and UPS measurements, are listed in Table 3. As expected, the HOMO–LUMO gap remained unaffected by side-chain deuteration, as observed in the optical measurements.^43^

**Table 3:** Summary of UV–Vis Spectroscopic Study and Ultraviolet Photoemission Spectroscopy (UPS) Study^b^^c^

Polymers	Mn (kDa)	Mw (kDa)	Absorbance λ_max_(nm)	*E*_g_^a^^a^ (eV)	HOMO (eV)	LUMO (eV)
D-DPP-T-C2C6C8	71.7	156	709, 793	1.25	–5.85	–4.57
H-DPP-T-C2C6C8	62.8	155	696, 783	1.26	–5.85	–4.59
D-DPP-T-C2C10C12	73.0	21.0	631, 706	1.25	–5.85	–4.59
H-DPP-T-C2C10C12	18.0	39.1	629, 703	1.25	–5.90	–4.65

a Bandgap was calculated from UV–vis measurements.

b HOMO was obtained from UPS measurements.

c LUMO was calculated using UV–vis spectroscopic measurements and UPS study. The molecular weight of the polymers listed here are different, indicating that the batches of DPP polymers used for the UPS and UV–vis study were distinct from those used in other characterizations.

Organic field-effect transistors (OFETs) of all DPPs were fabricated by using a TC-BG architecture to assess the impact of deuteration on their charge transport properties. The transfer curve of the devices based on DPP polymers is shown in Figure 3f, with full transfer and output characteristics provided in Figure S15. The charge carrier mobility values are summarized in Table 4 for the sample processed with thermal annealing at 150 °C for 1 h after spin coating. The charge mobilities were averaged from 17 to 19 devices per sample, yielding high mobilities of 1.51 × 10^–1^ and 1.86 × 10^–1^ cm^2^/V·s for deuterated and protonated DPP-T-C2C6C8, respectively. High charge mobilities are typically attributed to intermolecular interactions facilitated by interchain D–A interactions, which reduce π–π stacking distances and enhance molecular self-assembly.^70,71^ Here, X-ray scattering studies showed that side-chain deuteration did not affect the π–π stacking distance, suggesting that deuteration does not influence the charge transport properties of these polymers. However, as the side-chain length increased from C6C8 to C10C12, the mobilities decreased significantly. D-DPP-T-C2C10C12 and H-DPP-T-C2C10C12 exhibited charge mobilities of 5.5 × 10^–3^ and 5.9 × 10^–3^ cm^2^/V·s, respectively. Previous studies by Lipomi et al. and others have reported that increasing the side-chain length in low-bandgap polymers generally increases charge mobilities.^62,72^ However, various factors, such as morphology, device geometry, and molecular weight, can influence charge transport in polymers.^71^ We conclude here that there are few changes in the optoelectronic properties (absorption and charge carrier mobility) with deuteration of the alkyl side chains.

**Table 4:** Summary of OFET Device Measurements

Polymer	Average mobility, *μ*_avg_ (cm^2^/V·s)	Max mobility, *μ*_max_ (cm²/V·s)	*I*_on/off_
D-DPP-T-C2C6C8	1.51 × 10^–1^ ± 0.39 × 10^–1^	5.79 × 10^–1^	∼10^3^
H-DPP-T-C2C6C8	1.86 × 10^–1^ ± 0.40 × 10^–1^	7.00 × 10^–1^	∼10^3^
D-DPP-T-C2C10C12	5.50 × 10^–3^ ± 0.70 × 10^–3^	1.37 × 10^–2^	∼10^2^
H-DPP-T-C2C10C12	5.90 × 10^–3^ ± 0.14 × 10^–3^	2.58 × 10^–2^	∼10^2^

## Conclusion

Side-chain deuterated conjugated polymers were synthesized, and their effects on the thermal, crystalline, and optoelectronic properties were systematically examined. The melting and crystallization peaks were analyzed further using Gaussian fitting to determine the full width at half-maximum (FWHM). Thermal analysis revealed a significant increase in the melting and crystallization temperatures of DPP polymers after deuteration. However, investigations into molecular packing and optoelectronic properties, conducted using UPS, UV–vis spectroscopy, and thin-film transistor measurements, showed minimal isotopic effects. While earlier studies on D–A polymers using neutron scattering often overlooked isotopic influences, this research highlights the need for careful consideration of deuteration effects, particularly in thermal behavior.

## Supporting Information Available

Experimental details including chemical structure, dynamic mechanical analysis, thin films and solution UV–vis absorption spectra, and transistor measurements for DPP polymers (PDF)
