# Supplementary material for: Deuteration Effects on the Physical and Optoelectronic Properties of Donor–Acceptor Conjugated Polymers
Source: Macromolecules. 2025 Apr 29;58(9):4780–9. doi: 10.1021/acs.macromol.4c02778 (PMC12080320; doi:10.1021/acs.macromol.4c02778)
Supplement: Supplementary file 1 — ma4c02778_si_001.pdf [file ma4c02778_si_001.pdf]

Deuteration Effects on Physical and Optoelectronic Properties of Donor-Acceptor Conjugated Polymers

*Kundu Thapa[1], Madison Mooney[2], Guorong Ma[1], Zhiqiang Cao[1], Gage T. Mason[2], Naresh Eduguorala[3], Surabhi Jha[1], Derek L. Patton[1], Jason D. Azoulay[3], Simon Rondeau-Gagné[2], Xiaodan Gu[1]**

[1]School of Polymer Science and Engineering, The University of Southern Mississippi, Hattiesburg, Mississippi 39406, United States

[2]Department of Chemistry and Biochemistry, University of Windsor, Ontario N9B 3P4, Canada

[3]School of Chemistry and Biochemistry, Georgia Institution of Technology, Atlanta, Georgia 30332-0002, United States

KEYWORDS: conjugated polymer, deuteration effect, optoelectronic property

*Email: Xiaodan.gu@usm.edu

Contents

S1. Chemical structures of polymers. (a) DPP-T-C2C6C8 (b) DPP-T-C2C10C12. Synthesis protocol for deuterated side-chains and deuterated DPP polymers is reported in our previous literature.[1,2] 3

S2. DSC plots for repeated trials (A, B, C) of DPP polymers. 3

S3. Table summarizing repeated thermal measurements for DPP polymers, including the mean and standard deviation. The melting temperature is determined from the onset of the peak 3

S4. Table summarizing repeated thermal measurements for DPP polymers, including the mean and standard deviation. The crystallization temperature is reported based on the peak values. 3

S5. Fitting of T_m and T_c peaks from the DSC plots of the first repetition (A). 4

S6. Fitting of T_m and T_c peaks from the DSC plots of the second repetition (B). 4

S7. Fitting of T_m and T_c peaks from the DSC plots of the third repetition (C). 5

S8. Table summarizing the average FWHM for melting and crystallization peaks fitted using a Gaussian function in OriginPro software. 5

S9. Dynamic mechanical analysis (DMA) of deuterated (a,c) and protonated (b,d) DPP polymers. The protonated DPP polymers results are obtained from our previous work.[3] Tanδ peak is noted as the glass transition temperature. 6

S10. Table summary of AFM mean RMS roughness. 6

S11. AFM height and phase images for all DPP polymers, including DPP-T-C2C6C8 and DPP-T-C2C10C12, are presented. 7

S12. UV-Vis spectra for DPP thin films and solutions for both H/D DPP-T-C2C6C8 (a) and DPP-T-C2C10C12 (b) at room temperature in chlorobenzene. DPPs samples were not fully solubilized to form single chain at room temperature and usually dissolves at higher temperature (130 °C).[4] 7

S13. UV-Vis spectra processed for estimation of bandgap of by line extrapolation of the slope of the peak using Tauc Plot method.[5] The thickness of films were betweeen 40 to 90 nm. 8

S14. Table summary of UV-Vis spectroscopic study. The bandgap was estimated by linear extrapolation from the UV-Vis signal. 8

S15. Thin-film transistor measurements studied with top contact/bottom gate (TCBG) configuration for deuterated and protonated DPP polymers. The transfer characteristics (a) and output characteristics (b) of all the DPP polymers. 9

References 9

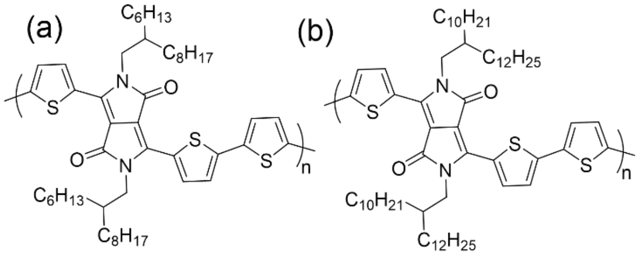

S1. Chemical structures of polymers. (a) DPP-T-C2C6C8 (b) DPP-T-C2C10C12. Synthesis protocol for deuterated side-chains and deuterated DPP polymers is reported in our previous literature.[1,2]

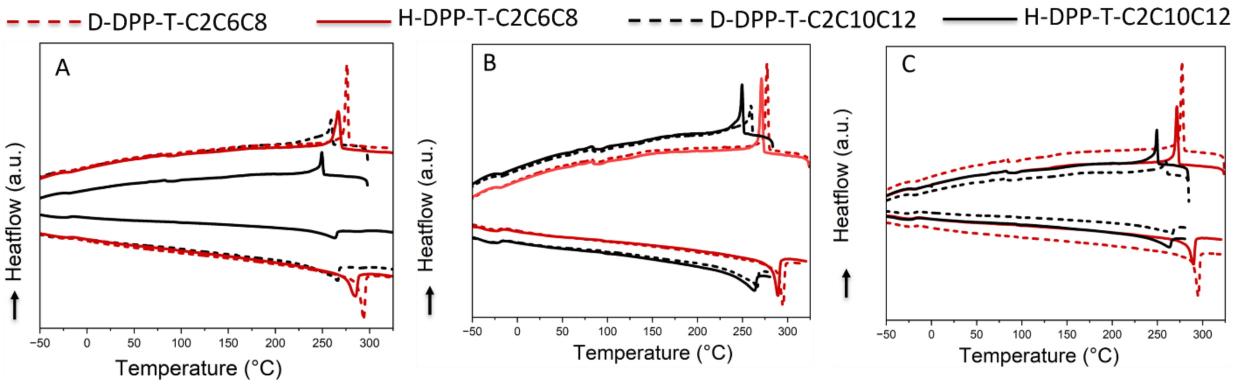

S2. DSC plots for repeated trials (A, B, C) of DPP polymers.

S3. Table summarizing repeated thermal measurements for DPP polymers, including the mean and standard deviation. The melting temperature is determined from the onset of the peak.

Melting temperature (°C)	1st	2nd	3rd	Average	Standard deviation
D-DPP-T-C2C6C8	289.6	289.6	290.2	289.0	1.5
H-DPP-T-C2C6C8	273.9	282.6	283.6	280.1	5.3
D-DPP-T-C2C10C12	251.8	252.4	254.0	252.7	1.1
H-DPP-T-C2C10C12	240.6	243.8	245.5	243.3	2.5

S4. Table summarizing repeated thermal measurements for DPP polymers, including the mean and standard deviation. The crystallization temperature is reported based on the peak values.

Crystallization temperature (°C)	1st	2nd	3rd	Average	Standard deviation
D-DPP-T-C2C6C8	276.1	277.3	277.2	276.9	0.7
H-DPP-T-C2C6C8	266.9	271.1	271.6	269.8	2.6
D-DPP-T-C2C10C12	259.3	259.3	259.3	259.3	0.1

H-DPP-T-C2C10C12	249.4	249.5	249.4	249.4	0.1

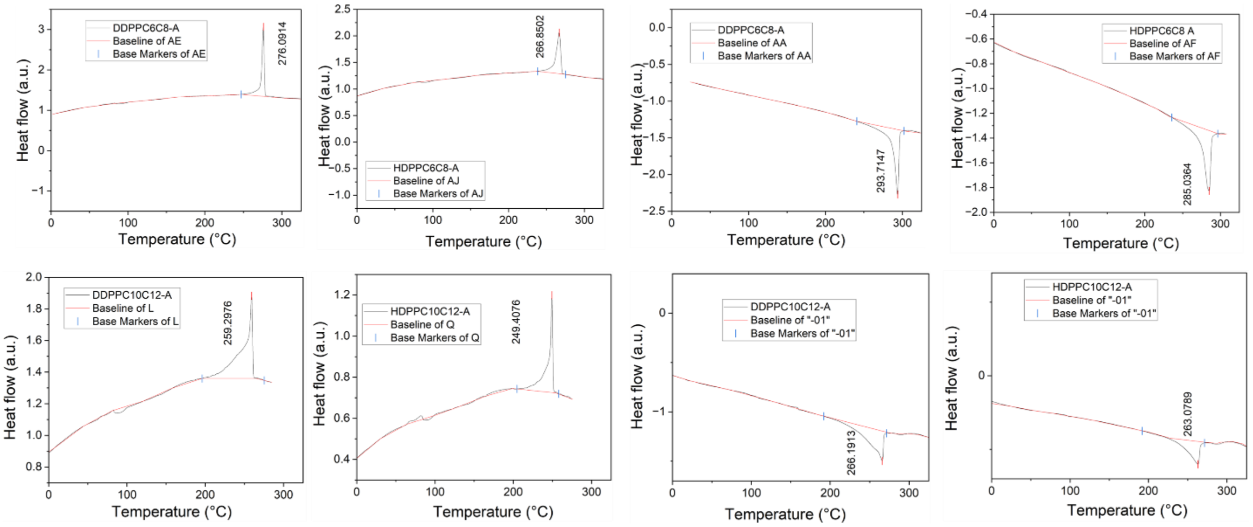

S5. Fitting of T_m and T_c peaks from the DSC plots of the first repetition (A).

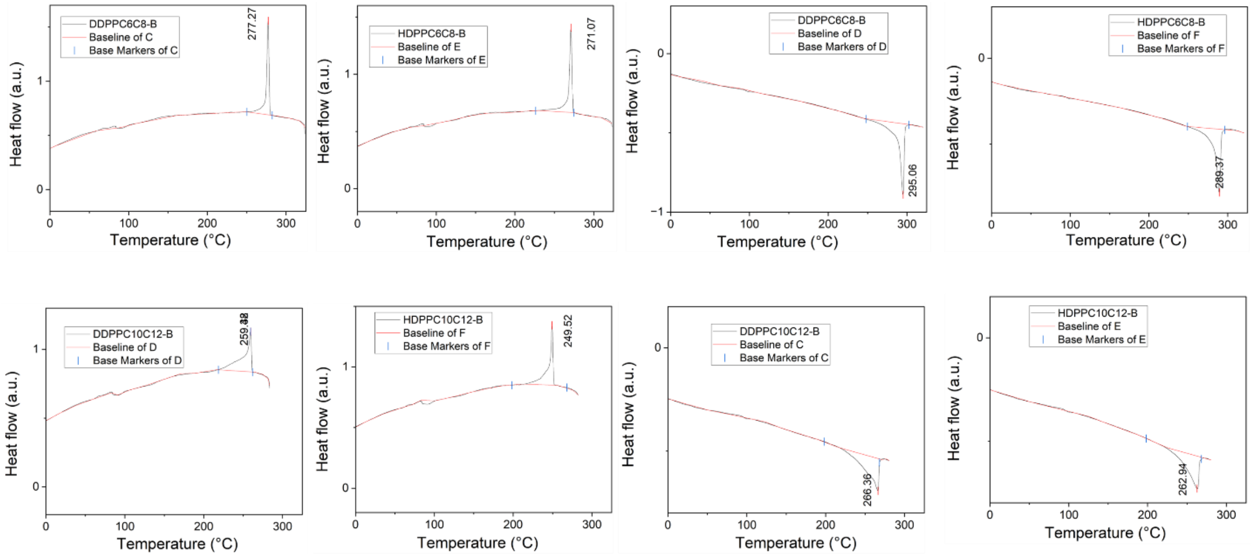

S6. Fitting of T_m and T_c peaks from the DSC plots of the second repetition (B).

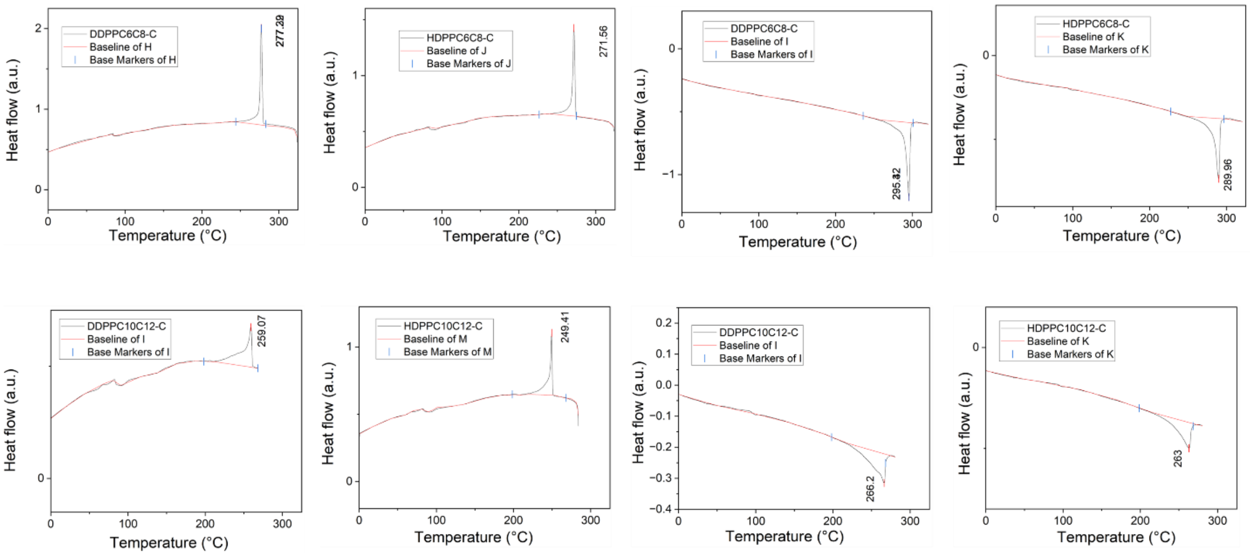

S7. Fitting of T_m and T_c peaks from the DSC plots of the third repetition (C).

S8. Table summarizing the average FWHM for melting and crystallization peaks fitted using a Gaussian function in OriginPro software.

FWHM (^{o}C)	T_m	T_c
D-DPP-T-C2C6C8	4.6 ± 1.2	2.51 ± 0.9
H-DPP-T-C2C6C8	6.7 ± 1.9	4.14 ± 1.8
D-DPP-T-C2C10C12	18.9 ± 3.5	5.2 ± 1.4
H-DPP-T-C2C10C12	15.5 ± 0.8	2.7 ± 0.4

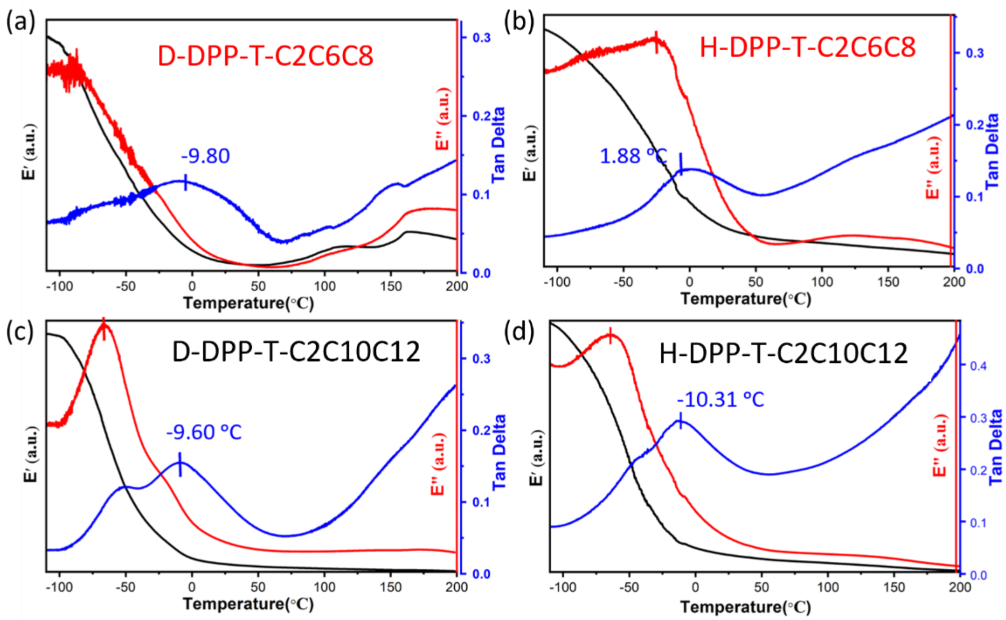

S9. Dynamic mechanical analysis (DMA) of deuterated (a,c) and protonated (b,d) DPP polymers. The protonated DPP polymers results are obtained from our previous work.[3] Tanδ peak is noted as the glass transition temperature.

S10. Table summary of AFM mean RMS roughness.

	Mean RMS Roughness (Å)
D-DPP-T-C2C6C8	1.89 ± 0.16
H-DPP-T-C2C6C8	2.31 ± 0.21
D-DPP-T-C2C10C12	0.55 ± 0.05
H-DPP-T-C2C10C12	0.61 ± 0.04

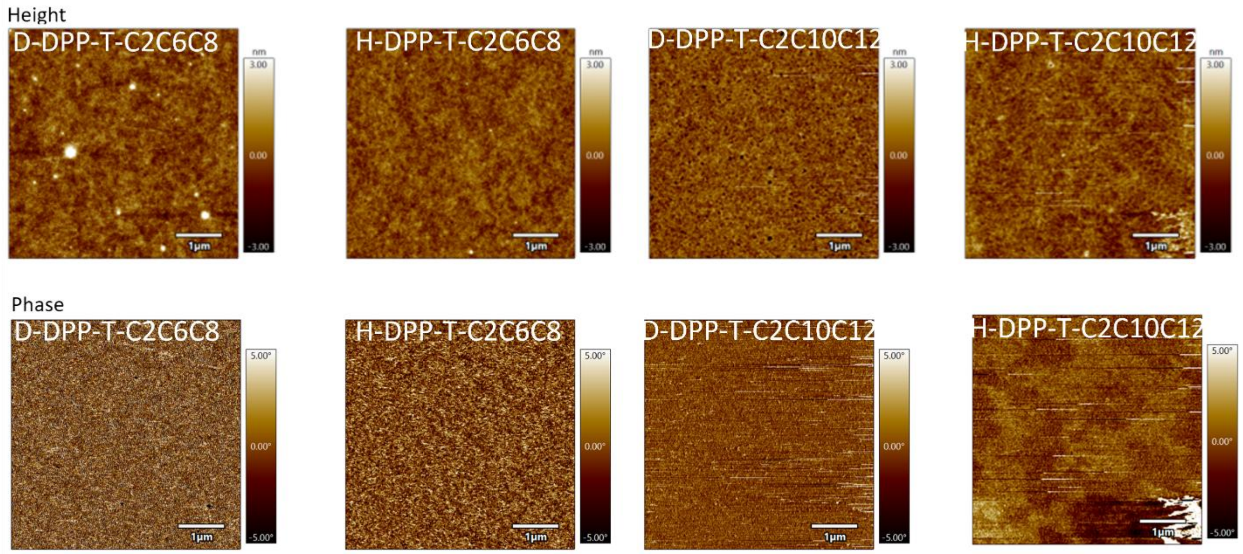

S11. AFM height and phase images for all DPP polymers, including DPP-T-C2C6C8 and DPP-T-C2C10C12, are presented.

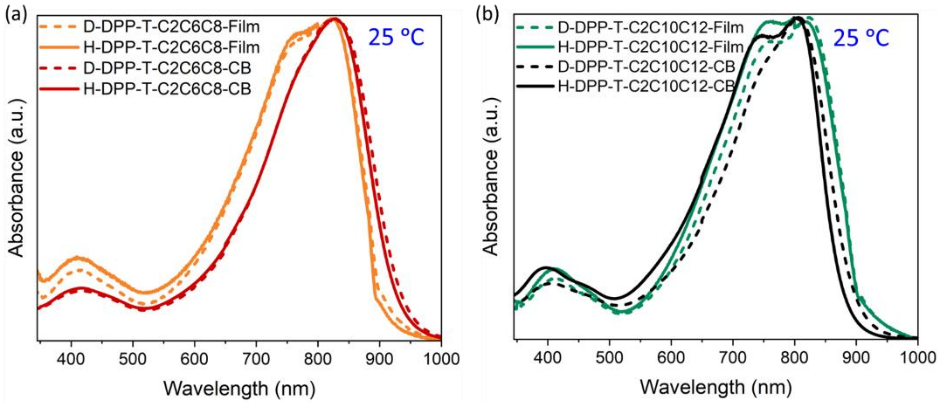

S12. UV-Vis spectra for DPP thin films and solutions for both H/D DPP-T-C2C6C8 (a) and DPP-T-C2C10C12 (b) at room temperature in chlorobenzene. DPPs samples were not fully solubilized to form single chain at room temperature and usually dissolves at higher temperature (130 °C).[4]

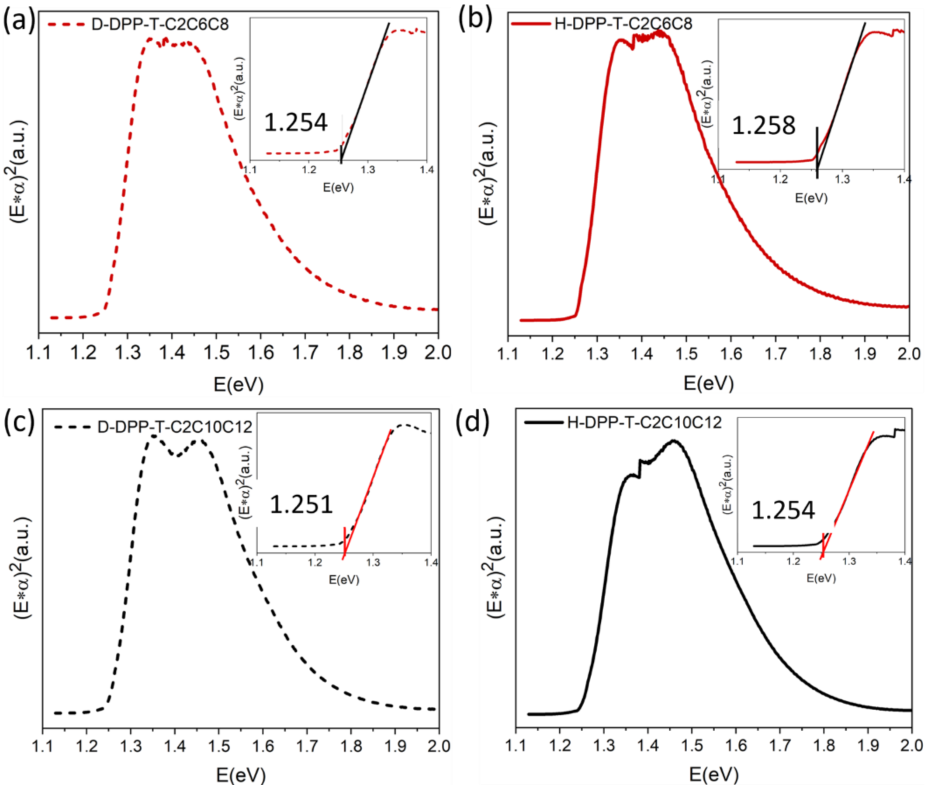

S13. UV-Vis spectra processed for estimation of bandgap of by line extrapolation of the slope of the peak using Tauc Plot method.[5] The thickness of films were between 40 to 90 nm.

S14. Table summary of UV-Vis spectroscopic study. The bandgap was estimated by linear extrapolation from the UV-Vis signal.

	Films λ (nm^{-1})	Solution λ (nm^{-1})	Bandgap (eV)
D-DPP-T-C2C6C8	745, 832	769, 847	1.25
H-DPP-T-C2C6C8	744, 830	768, 842	1.26
D-DPP-T-C2C10C12	760, 842	760, 822	1.25
H-DPP-T-C2C10C12	763, 839	739, 813	1.25

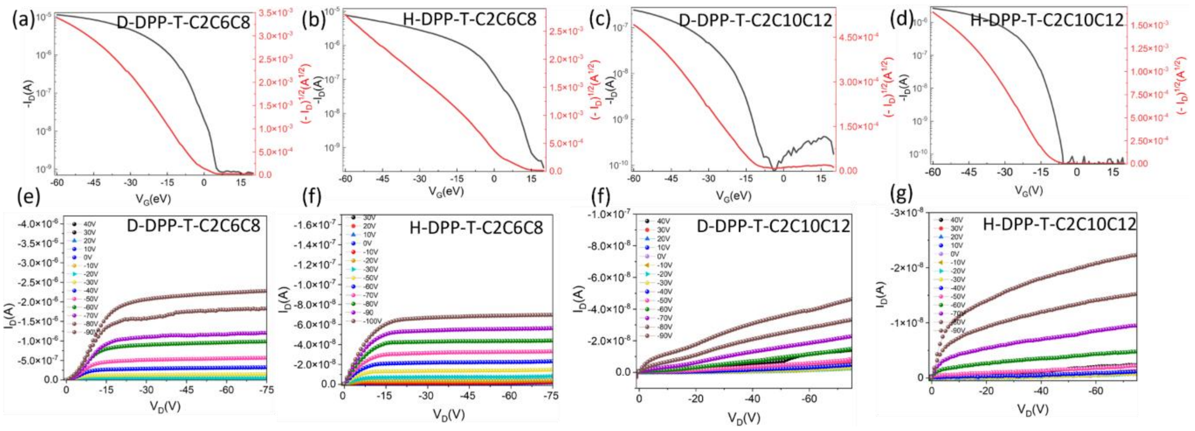

S15. Thin-film transistor measurements studied with top contact/bottom gate (TCBG) configuration for deuterated and protonated DPP polymers. The transfer characteristics (a) and output characteristics (b) of all the DPP polymers.

References

(1) Cao, Z.; Li, Z.; Zhang, S.; Galuska, L.; Li, T.; Do, C.; Xia, W.; Hong, K.; Gu, X. Decoupling Poly(3-Alkylthiophenes)' Backbone and Side-Chain Conformation by Selective Deuteration and Neutron Scattering. *Macromolecules* **2020**, *53* (24), 11142–11152, DOI: 10.1021/acs.macromol.0c02086

(2) Cao,Z.;Li, Z.; Mooney, M.; Do,C.; Hong, K.; Rondeau-Gagné, S.; Xia, W.; Gu, X. Uncovering Backbone Conformation for Rigid DPP-Based Donor-Acceptor Conjugated Polymer Using Deuterium Labeling and Neutron Scattering. *Macromol. Under Rev.* **2024**, 57, 10379-10388

(3) Zhang, S.; Alesadi, A.; Selivanova, M.; Cao, Z.; Qian, Z.; Luo, S.; Galuska, L.; Teh, C.; Ocheje, M. U.; Mason, G. T.; St. Onge, P. B. J.; Zhou, D.; Rondeau-Gagné, S.; Xia, W.; Gu, X. Toward the Prediction and Control of Glass Transition Temperature for Donor–Acceptor Polymers. *Adv. Funct. Mater.* **2020**, *30* (27), DOI: 10.1002/adfm.202002221

(4) Cao, Z.; Li, Z.; Tolba, S. A.; Mason, G. T.; Xiong, M.; Ocheje, M. U.; Alesadi, A.; Do, C.; Hong, K.; Lei, T.; Rondeau-Gagné, S.; Xia, W.; Gu, X. Probing Single-Chain Conformation and Its Impact on the Optoelectronic Properties of Donor–Accepter Conjugated Polymers. *J. Mater. Chem. A* **2023**, *11* (24), 12928–12940, DOI: 10.1039/D2TA09389H

(5) Jain, S. K.; Kumar, R. R.; Aggarwal, N.; Vashishtha, P.; Goswami, L.; Kuriakose, S.; Pandey, A.; Bhaskaran, M.; Walia, S.; Gupta, G. Current Transport and Band Alignment Study of MoS 2 /GaN and MoS 2 /AlGaN Heterointerfaces for Broadband Photodetection Application. *ACS Appl. Electron. Mater.* **2020**, *2* (3), 710–718, DOI: 10.1021/acsaelm.9b00793
